# Apoc2 loss-of-function zebrafish mutant as a genetic model of hyperlipidemia

**DOI:** 10.1242/dmm.019836

**Published:** 2015-08-01

**Authors:** Chao Liu, Keith P. Gates, Longhou Fang, Marcelo J. Amar, Dina A. Schneider, Honglian Geng, Wei Huang, Jungsu Kim, Jennifer Pattison, Jian Zhang, Joseph L. Witztum, Alan T. Remaley, P. Duc Dong, Yury I. Miller

**Affiliations:** 1 Division of Endocrinology and Metabolism, Department of Medicine, University of California, San Diego, La Jolla, CA, USA; 2 Sanford Children's Health Research Center, Programs in Genetic Disease and Development and Aging, and Stem Cell and Regenerative Biology, Sanford-Burnham Medical Research Institute, La Jolla, CA, USA; 3 Lipoprotein Metabolism Section, Cardiopulmonary Branch, NHLBI, NIH, Bethesda, MD, USA; 4 State Key Laboratory of Molecular Developmental Biology, Institute of Genetics and Developmental Biology, Chinese Academy of Sciences, Beijing, China

**Keywords:** Zebrafish, Apolipoprotein C-II, APOC2, Lipoprotein lipase, Hyperlipidemia

## Abstract

Apolipoprotein C-II (APOC2) is an obligatory activator of lipoprotein lipase. Human patients with APOC2 deficiency display severe hypertriglyceridemia while consuming a normal diet, often manifesting xanthomas, lipemia retinalis and pancreatitis. Hypertriglyceridemia is also an important risk factor for development of cardiovascular disease. Animal models to study hypertriglyceridemia are limited, with no *Apoc2*-knockout mouse reported. To develop a genetic model of hypertriglyceridemia, we generated an *apoc2* mutant zebrafish characterized by the loss of Apoc2 function. *apoc2* mutants show decreased plasma lipase activity and display chylomicronemia and severe hypertriglyceridemia, which closely resemble the phenotype observed in human patients with APOC2 deficiency. The hypertriglyceridemia in *apoc2* mutants is rescued by injection of plasma from wild-type zebrafish or by injection of a human APOC2 mimetic peptide. Consistent with a previous report of a transient *apoc2* knockdown, *apoc2* mutant larvae have a minor delay in yolk consumption and angiogenesis. Furthermore, *apoc2* mutants fed a normal diet accumulate lipid and lipid-laden macrophages in the vasculature, which resemble early events in the development of human atherosclerotic lesions. In addition, *apoc2* mutant embryos show ectopic overgrowth of pancreas. Taken together, our data suggest that the *apoc2* mutant zebrafish is a robust and versatile animal model to study hypertriglyceridemia and the mechanisms involved in the pathogenesis of associated human diseases.

## Abstract (precis)

**Highlighted Article:** Apoc2 loss-of-function zebrafish display severe hypertriglyceridemia, which is characteristic of human patients with defective lipoprotein lipase activity.

## INTRODUCTION

Hypertriglyceridemia is an independent risk factor for cardiovascular disease (CVD) (Do et al., 2013) and is positively associated with obesity, insulin resistance, type 2 diabetes and other metabolic syndromes (Watts et al., 2013). Hypertriglyceridemia is the result of interactions between non-genetic and genetic factors. The most common non-genetic factors causing hypertriglyceridemia are obesity, alcohol excess, lack of exercise and unhealthy diets (Miller et al., 2011; Watts et al., 2013). Genetic factors account for 50% of individual variation in plasma triglyceride (TG) levels (Goldberg et al., 2011; Miller et al., 2011; Namboodiri et al., 1985), whereas severe hypertriglyceridemia (TG>885 mg/dl) is usually caused by single mutations in the gene encoding lipoprotein lipase (LPL), or less commonly, in genes affecting proteins involved in LPL activity, such as apolipoprotein C-II (APOC2), lipase maturation factor 1 (LMF1), apolipoprotein A-V (APOA5) and GPI HDL binding protein1 (GPIHBP1) (Surendran et al., 2012).

APOC2 is an obligatory co-activating factor for LPL, which is the key enzyme responsible for hydrolysis of plasma TG (Fukushima and Yamamoto, 2004; Kei et al., 2012). Patients with APOC2 or LPL deficiency show severe hypertriglyceridemia and chylomicronemia, and often manifest eruptive xanthomas, lipemia retinalis and acute and recurrent pancreatitis, which can be lethal (Breckenridge et al., 1978; Cox et al., 1978; Ewald et al., 2009; Goldberg and Merkel, 2001; Miller et al., 2011; Scherer et al., 2014; Watts et al., 2013). Current genetic animal models to study hyperlipidemia include mice with a conditional deficiency in LPL, deficiency of GPIHBP1, or with overexpression of human APOC2 and APOC3 (Ebara et al., 1997; Goulbourne et al., 2014; Shachter et al., 1994; Weinstein et al., 2010). There are several systemic or tissue specific *Lpl*-knockout mouse models that have been developed (Garcia-Arcos et al., 2013; Merkel et al., 1998; Trent et al., 2014; Weinstock et al., 1995). The systemic *Lpl*-knockout mice develop hypertriglyceridemia but die shortly after birth. The neonatal death, together with the hypertriglyceridemia, are prevented by overexpression of human LPL in either skeletal or cardiac muscle (Levak-Frank et al., 1999; Weinstock et al., 1995). In contrast to extensive work conducted with *Lpl-*knockout mice, *Apoc2*-knockout mouse models have not been reported.

Zebrafish is an emerging animal model to study lipid metabolism and mechanisms of human disease related to lipid abnormalities (Anderson et al., 2011; Fang et al., 2014; Holtta-Vuori et al., 2010; Levic et al., 2015; O'Hare et al., 2014; Stoletov et al., 2009). The advantages of using zebrafish include large progeny numbers, optical transparency of zebrafish larvae, easy genetic manipulation and cost-effective maintenance (Dooley and Zon, 2000). Importantly, genes involved in lipid and lipoprotein metabolism, such as *APOB*, *APOE*, *APOA1*, *LDLR*, *APOC2*, *LPL*, *LCAT* and *CETP*, are conserved from zebrafish to humans (Fang et al., 2014; Holtta-Vuori et al., 2010; Otis et al., 2015). Specifically, the zebrafish Lpl (NCBI Gene ID: 30354) amino acid sequence is 61.7% identical to its human ortholog, with 79.7% conserved consensus amino acids. The zebrafish full-length Apoc2 (NCBI Gene ID: 568972, molecular mass 11.2 kDa) is only 27.8% identical to human APOC2, with 49.5% conserved consensus amino acids, but its C-terminal region (residues 67-92, zebrafish numbering), which mediates Apoc2 binding to Lpl, shows 46.1% identity to the LPL-binding region of human APOC2. Thus, it is likely that the APOC2-LPL complex required for TG hydrolysis is also conserved in zebrafish. To support this hypothesis, we have developed an Apoc2 loss-of-function zebrafish model. Our results demonstrate that the *apoc2* mutant zebrafish develop severe hypertriglyceridemia, which is characteristic for human patients deficient in APOC2, and that the *apoc2* mutant is a suitable animal model to study hyperlipidemia and the mechanisms involved in the pathogenesis of associated diseases.

**RESOURCE IMPACT**

**Background**

Blood transports dietary lipids and the lipids produced by the liver around the body in the form of large lipoprotein particles, such as chylomicrons and very-low-density lipoproteins (VLDL). These lipoproteins also carry APOC2, a protein that is needed to activate lipoprotein lipase (LPL), which hydrolyzes triglycerides to deliver fatty acids to tissues. Hypertriglyceridemia (high levels of triglycerides in the blood) is an independent risk factor for cardiovascular disease and is positively associated with metabolic disorders. Obesity, alcohol excess, lack of exercise and an unhealthy diets can all cause hypertriglyceridemia but, in addition, patients with a genetic defect in APOC2 or LPL have severe hypertriglyceridemia and often manifest skin and eye abnormalities (eruptive xanthomas and lipemia retinalis, respectively) and acute and recurrent pancreatitis, which can be lethal. The mechanisms that underlie hypertriglyceridemia-associated diseases are not well understood, in part, owing to the limited availability of animal models. Specifically, although substantial work has been conducted with *Lpl*-knockout mice, *Apoc2*-knockout mouse models have not been reported.

**Results**

Zebrafish is an emerging animal model for the study of lipid metabolism and the mechanisms of human disease related to lipid abnormalities. The advantages of using zebrafish include large progeny numbers, optical transparency of zebrafish larvae, easy genetic manipulation and cost-effective maintenance. In this study, the authors generate an *apoc2* mutant zebrafish characterized by the loss of Apoc2 function. The authors report that Apoc2 loss-of-function mutant zebrafish display chylomicronemia (build-up of chylomicrons in the blood) and severe hypertriglyceridemia, characteristics that closely resemble those seen in human patients with APOC2 deficiency. They show that the hypertriglyceridemia in *apoc2* mutants can be rescued by injection of plasma from wild-type zebrafish with functional Apoc2 or by injection of a human APOC2 mimetic peptide. Notably, *apoc2* mutants fed a normal diet accumulate lipid and lipid-laden macrophages in the vasculature, which resembles early events in the development of human atherosclerotic lesions. Finally, *apoc2* mutant embryos show ectopic overgrowth of pancreas.

**Implications and future directions**

Taken together, these findings indicate that the new *apoc2* mutant zebrafish generated by the authors display a robust hyperlipidemia phenotype and could, therefore, be a useful and versatile animal model in which to study the mechanisms that underlie the human diseases induced by hypertriglyceridemia. Moreover, small molecule and genetic screens using the *apoc2* mutant zebrafish might suggest new approaches to treatment of hyperlipidemia and related diseases.

## RESULTS

### Mutation of zebrafish *apoc2* gene with TALENs

**Fig. 1.**
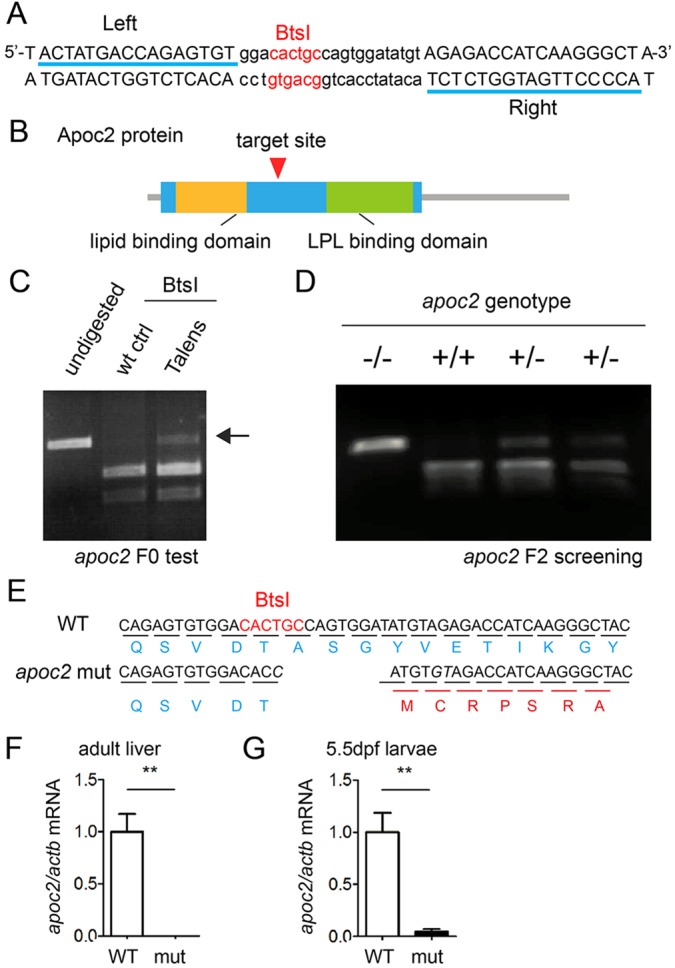
**Generation of *apoc2* mutant zebrafish.** (A) A diagram of partial *apoc2* genomic DNA sequence, showing the TALEN-binding sites (underlined) and the spacer DNA (lowercase), which contains the BtsI restriction site. (B) A diagram of the TALEN target site in the Apoc2 protein. (C) Test for TALEN mutagenesis in F0 zebrafish with BtsI enzyme digestion. The arrow indicates the modified genomic DNA, which was no longer recognized and cut by BtsI. (D) Representative genotyping results of the F2 generation. (E) Sequencing data suggest a frame shift mutation at the site preceding the LPL-binding domain in the *apoc2* mutant. (F,G) *apoc2* mRNA expression (as assessed by qPCR) in adult liver and 5.5 dpf larvae. Results are mean±s.e.m.; *n*=3 in each group. **P<0.01 (Student's *t*-test).

To create a zebrafish model of hypertriglyceridemia, we mutated the *apoc2* gene in zebrafish using a transcription activator-like effector nuclease (TALEN) technique. We chose a target site located at exon 3 of the *apoc2* gene, with TALEN-binding sequences of 16 bp and 17 bp nucleotides on the left and right side of the target site, respectively. The spacer DNA was 21 bp long and contained the BtsI restriction enzyme site (Fig. 1A). The corresponding protein coding region of the TALEN target site is located in front of the Lpl-binding domain (Fig. 1B). The mRNAs encoding TALENs were injected into one-cell stage zebrafish embryos. To test whether the *apoc2* TALEN pair disrupted the *apoc2* gene at its specific target site, we extracted genomic DNA from F0 generation zebrafish, amplified the regions containing the target site with PCR and conducted BtsI enzyme digestion. Compared with wild-type (WT), a part of the PCR band amplified from TALEN-injected F0 zebrafish was resistant to enzyme digestion (Fig. 1C), consistent with modification of the BtsI recognition site in the *apoc2* genomic sequence. Then, F0 zebrafish were outcrossed with WT zebrafish and the F1 progeny were genotyped and outcrossed again for two generations, followed by an incross to obtain homozygous mutants (Fig. 1D). Sequencing of the *apoc2* mutant revealed a one-nucleotide replacement and ten-nucleotide deletion, resulting in a frame shift mutation changing the coding sequence of the Lpl-binding domain of the Apoc2 protein (Fig. 1E).

We then isolated total RNA from the *apoc2* mutant adult liver or 5.5 days post fertilization (dpf) larvae and conducted quantitative real-time PCR (qPCR) with primers specific to both WT and mutated *apoc2* mRNA. The *apoc2* mRNA was undetectable in the adult liver of the mutant (Fig. 1F) and dramatically decreased in 5.5 dpf larvae (Fig. 1G). These data suggest that *apoc2* mRNA transcripts that contain the frame shift are eliminated, as has been demonstrated for many other premature stop codon and frame shift transcripts (Baker and Parker, 2004), and suggest that the mutation might result in loss of Apoc2 function.

### Hyperlipidemia in adult *apoc2* mutants

**Fig. 2.**
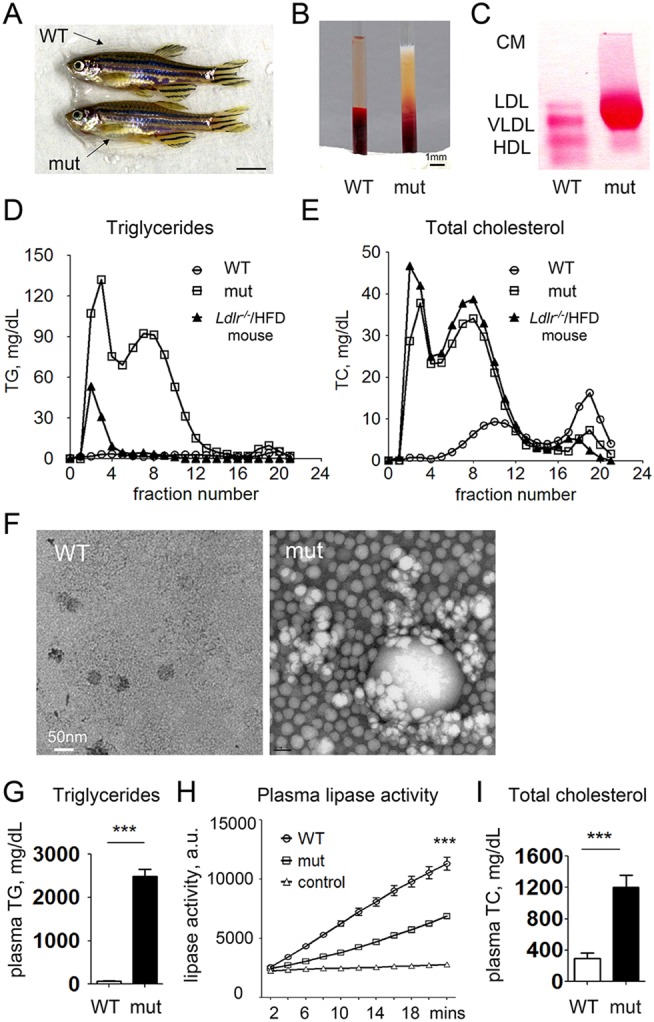
**Adult *apoc2* mutant zebrafish develop hyperlipidemia when fed a normal diet.** (A) No apparent differences between 4-month-old male WT and *apoc2* mutant (mut) zebrafish. Scale bar: 0.5 cm. (B) Clear and milky appearance of plasma from WT and *apoc2* mutant zebrafish, respectively. (C) Native gel electrophoresis and neutral lipid staining of plasma from WT and *apoc2* mutant zebrafish. CM, chylomicron. (D,E) Fast protein liquid chromatography (FPLC) lipoprotein profile of pooled WT (45 animals) and *apoc2* mutant (14 animals) zebrafish plasma. Plasma from an *Ldlr^−/−^* mouse fed a 60% high-fat diet (HFD) was used for comparison. (F) Electron microscopy images of TRLs isolated from pooled WT and *apoc2* mutant zebrafish plasma by ultracentrifugation as described in the Materials and Methods. (G) Plasma TG levels. Results are mean±s.e.m.; *n*=6 in each group; ***P<0.001 (Student's *t*-test). (H) Plasma lipase activity assayed as described in the Materials and Methods. Assay buffer was used as negative control. Results are mean±s.e.m.; *n*=3 in each group; ***P<0.001. (I) Plasma TC levels. Results are mean±s.e.m.; *n*=6 in each group; ***P<0.001 (Student's *t*-test).

Adult *apoc2* mutants were viable and fertile, and had a normal physical appearance (Fig. 2A and supplementary material Fig. S1). To test whether *apoc2* deficiency resulted in hypertriglyceridemia, we drew blood from adult male zebrafish into micro-capillary tubes at 8 h after the last feeding and let the capillaries stand overnight at 4°C to sediment erythrocytes. Plasma from an *apoc2* mutant showed a milky phenotype, with a creamy layer at the top (Fig. 2B), typical of that found in subjects with APOC2 or LPL deficiency (Baggio et al., 1986; Fellin et al., 1983). These plasma samples were also subjected to native agarose gel electrophoresis. Compared with WT, mutant plasma showed a decrease in HDL and a dramatic increase in the very-low-density lipoprotein (VLDL) and low-density lipoprotein (LDL) fraction. Chylomicrons, which were never observed in fasted WT zebrafish, were present in the mutant (Fig. 2C, gel origin). Size exclusion chromatography of pooled zebrafish plasma from *apoc2* mutants showed that there was a dramatic increase of TG and total cholesterol (TC) in the chylomicron and VLDL fraction, as well as in LDL fraction, but decreased TC in the high-density lipoprotein (HDL) fraction, compared with that in WT zebrafish (Fig. 2D,E). For comparison, plasma from an *Ldlr^−/−^* mouse fed a 60% high-fat diet showed a plasma lipoprotein profile similar to the *apoc2* mutant, although it had lower TG levels. Pooled plasma from overnight fasted zebrafish was also subjected to ultracentrifugation to float triglyceride-rich lipoproteins (TRLs). The uppermost layer, corresponding to TRLs, collected from WT and *apoc2* mutant zebrafish, was then subjected to electron microscopy to visualize lipoproteins. Although no TRLs were detected in the WT plasma, TRLs of different sizes, including large chylomicrons, were abundant in *apoc2* mutant plasma (Fig. 2F). Accordingly, plasma TG levels were dramatically increased (2488±160 mg/dl versus 67±8 mg/dl, mutant versus WT; mean±s.e.m., *n*=6, *P*<0.001, Fig. 2G) and plasma lipase activity was significantly decreased in *apoc2* mutants compared with WT (Fig. 2H). Although it has been reported that human patients with APOC2 deficiency have a mild elevation in plasma cholesterol (Baggio et al., 1986; Fellin et al., 1983), unexpectedly, plasma TC levels were increased as much as four-fold in *apoc2* mutants compared with WT zebrafish (1202±63 mg/dl versus 296±26 mg/dl, mutant versus WT; mean±s.e.m., *n*=6, *P*<0.001) (Fig. 2I).

Taken together, these data suggest that the TALEN-induced mutation in the *apoc2* gene results in severe hypertriglyceridemia and chylomicronemia, confirming that the function of APOC2 to activate LPL is conserved in zebrafish.

### Hyperlipidemia in *apoc2* mutant larvae

**Fig. 3.**
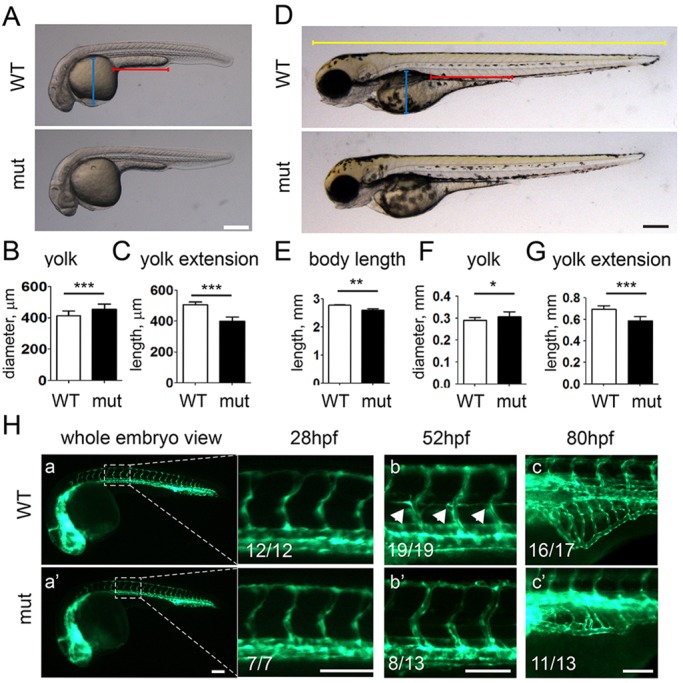
**Development and angiogenesis in *apoc2* mutant larvae.** (A-C) Morphology of WT (*n*=11) and *apoc2* mutant (mut, *n*=11) embryos at 30 hpf. Results are mean±s.e.m.; ***P<0.001 (Student's *t*-test). (D-G) Morphology of WT (*n*=11) and *apoc2* mutant (*n*=10) embryos at 3.5 dpf. Results are mean±s.e.m.; *P<0.05; **P<0.01; ***P<0.001 (Student's *t*-test). The blue, red and yellow lengths highlighted in D were used to delineate the size of yolk, yolk extension and the body length, respectively. (H) Angiogenesis in *fli1:EGFP* WT and *apoc2* mutant embryos at 28 hpf (a,a′), 52 hpf (b,b′) and 80 hpf (c,c′). The numbers in the figure indicate the number of animals exhibiting the illustrated phenotype and the total number of animals. Scale bars: 200 µm (A,D); 100 µm (H).

*apoc2* mutant embryos and larvae did not show significant defects in development, other than minor, but statistically significant, delays in yolk utilization and growth. At 30 h post fertilization (hpf) and 3.5 dpf, the yolk in the mutant was larger but the body length was shorter than that of the WT (Fig. 3A-G). However, the development stage reached at both 30 hpf and 3.5 dpf, which was determined by the head position and the pigment patterns, were similar in mutants and WT (Fig. 3A,D).

It has been reported that transient knockdown of *apoc2* inhibits angiogenesis (Avraham-Davidi et al., 2012). To assess embryonic angiogenesis, we crossed the *apoc2* mutant with *fli1-EGFP* transgenic zebrafish, which express EGFP in endothelial cells. At 26 hpf, there were no angiogenesis differences between *apoc2* mutant and WT zebrafish (Fig. 3Ha,a′). However, at 52 hpf, growth of inter-segmental blood vessels (ISV) was delayed in the *apoc2* mutant (Fig. 3Hb,b′), as was the outgrowth of subintestinal veins (SIV) at 80 hpf (Fig. 3Hc,c′), consistent with the results reported by Avraham-Davidi et al. Interestingly, these ISV and SIV defects in mutants were all corrected at later stages and the vasculature of 14 dpf mutants was normal (supplementary material Fig. S2).

**Fig. 4.**
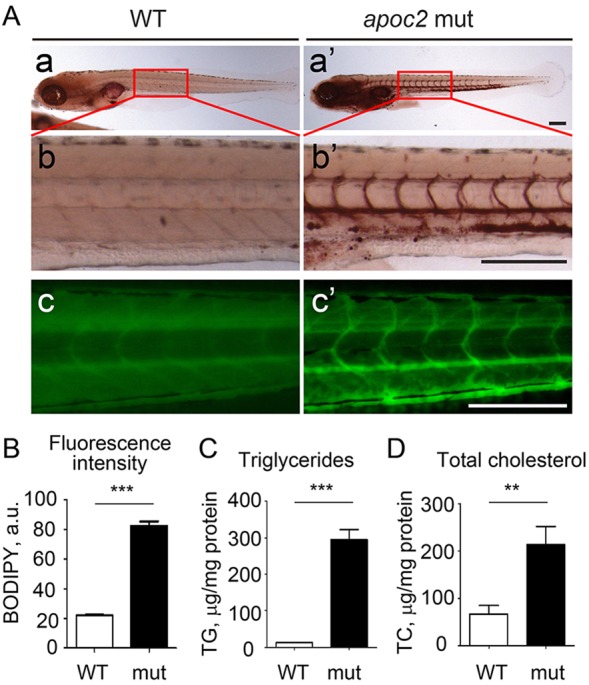
***apoc2* mutant larvae develop hyperlipidemia.** (A) Non-fed 6 dpf WT and *apoc2* mutant (mut) larvae were stained with Oil Red O (a-b′) or BODIPY (c,c′). Scale bar: 200 µm. (B) Quantification of BODIPY staining in A. Results are mean±s.e.m.; *n*=3 in each group; ***P<0.001 (Student's *t*-test). (C,D) TG and TC levels in homogenates of 6 dpf WT and *apoc2* mutant larvae, normalized to total protein content. Results are mean±s.e.m.; *n*=3 in each group; **P<0.01; ***P<0.001 (Student's *t*-test).

Until 5 dpf, zebrafish embryos, unlike adults, receive nutrients through yolk utilization. To test whether TG and cholesterol levels were affected at this early stage, which is a suitable time window for drug screening and genetic manipulation by injection of antisense morpholino oligonucleotides, we stained embryos at 1-6 dpf with Oil Red O (ORO), which stains neutral lipids, including TG and cholesterol esters (CEs). Before 3 dpf, the strongest ORO staining was in the yolk and no significant differences between WT and *apoc2* mutants were detected. Starting from 3 dpf, we noticed consistently stronger vascular ORO staining in the mutants, with the most significant differences between WT and *apoc2* mutants observed in non-fed 6 dpf embryos. At this stage, ORO staining was weak or absent in the blood vessels of WT zebrafish, but strong ORO staining was detected in the *apoc2* mutants (Fig. 4Aa,a′,b,b′). The dramatic differences in the levels of circulating neutral lipids were also monitored with the fluorescent dye BODIPY (Fig. 4Ac,c′). The advantage of using BODIPY is that live zebrafish can be immersed in the stain and after a wash, changes in neutral lipid levels can be monitored and quantified (Fig. 4B). Furthermore, the BODIPY staining in live zebrafish persisted over a prolonged period of time (up to 48 h in *apoc2* mutants in our experiments). Consistent with the staining results, TG and TC levels were significantly increased in the homogenates of 6 dpf *apoc2* mutant larvae compared with WT (Fig. 4C,D).

In addition to the *apoc2* mutant characterized in Figs 1-4, we generated a second zebrafish line with a different *apoc2* mutation, which introduced a stop codon in the target site, causing loss of the Lpl-binding domain of Apoc2. This *apoc2* mutant (line 2) also displayed increased vascular ORO and BODIPY staining at 6 dpf, similar to the frame shift mutant, and the loss of *apoc2* mRNA (supplementary material Fig. S3), further supporting the functional conservation of Apoc2 in zebrafish.

### Injection of normal plasma rescues hyperlipidemia in *apoc2* mutants

**Fig. 5.**
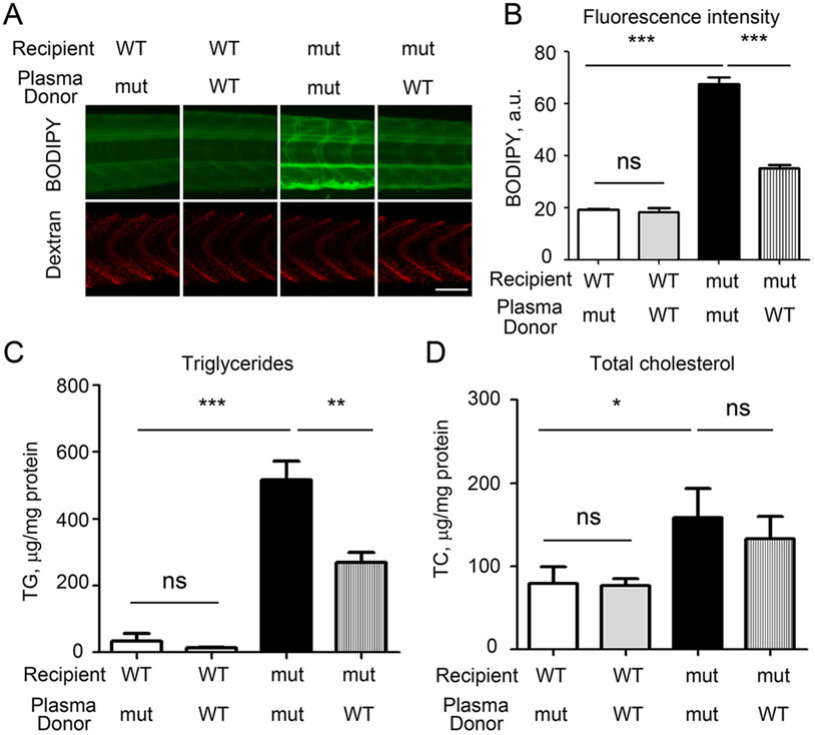
**Injections of WT zebrafish plasma rescue the hyperlipidemia phenotype in *apoc2* mutants.** (A) *apoc2* WT or mutant larvae at 5 dpf were injected with 5 nl of undiluted plasma from either WT or mutant (mut) adult male zebrafish, both supplemented with red fluorescent dextran. At 1 day after injection, at 6 dpf, larvae were stained with BODIPY (green fluorescence) to assess the neutral lipid content of plasma. Red dextran fluorescence indicates successful injection. Scale bar: 100 µm. (B) Quantification of results shown in A. Results are mean±s.e.m.; *n*=3 in each group; ***P<0.001; ns, not significant (Student's *t*-test). (C,D) TG and TC levels in homogenates of 6 dpf WT and *apoc2* mutant larvae, normalized to total protein content. Results are mean±s.e.m.; five embryos pooled per sample, *n*=3 in each group; *P<0.05; **P<0.01 (Student's *t*-test).

In human patients with APOC2 deficiency, transfusions of normal plasma result in rapid and dramatic decreases in the plasma TG levels in patients (Breckenridge et al., 1978; Nordestgaard et al., 1988). To test whether this effect can be reproduced in our *apoc2* mutant zebrafish, we isolated plasma from adult WT and mutant zebrafish and transfused them into WT and *apoc2* mutant larva recipients through cardinal vein injection. The plasma was supplemented with red fluorescent dextran as a tracer to confirm a successful injection. At 24 h after injection, the larvae with positive dextran fluorescence were selected and stained with BODIPY. The WT larvae injected with WT or mutant plasma did not show changes in BODIPY staining. Compared with *apoc2* mutant plasma-injected mutant larvae, WT plasma-injected *apoc2* mutant larvae showed a significant decrease in BODIPY fluorescence (Fig. 5A,B). Following BODIPY imaging, the same larvae were euthanized and homogenized, and lipids were measured. Consistent with the BODIPY-staining results, TG levels in the *apoc2* mutants were significantly reduced following the injection of WT plasma (Fig. 5C,D).

### Injection of APOC2 mimetic peptide rescues hyperlipidemia in *apoc2* mutants

**Fig. 6.**
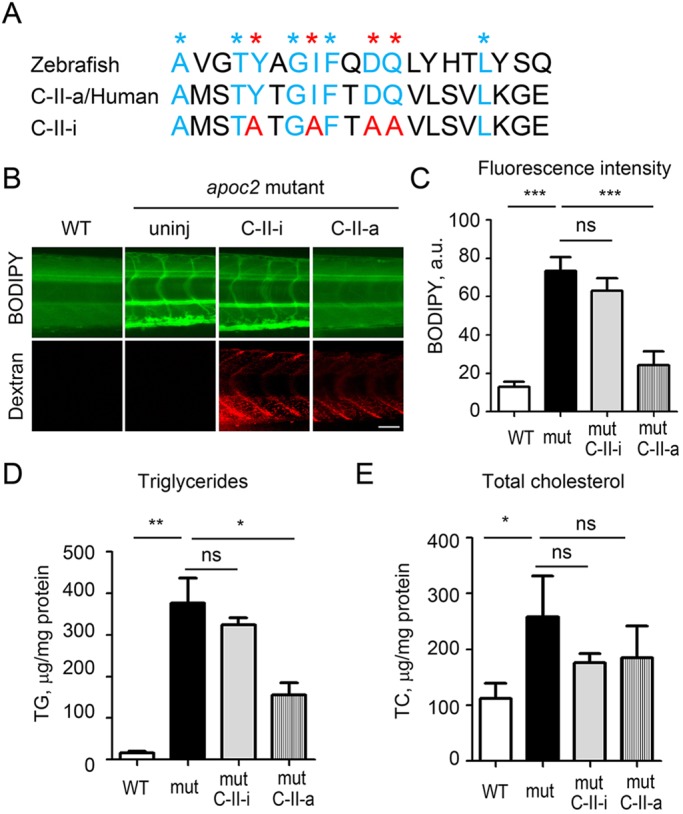
**Injections of human APOC2 mimetic peptide rescue hyperlipidemia phenotype in *apoc2* mutant.** (A) Protein alignment of APOC2 mimetic peptides with the corresponding zebrafish Apoc2 amino acids sequence. Blue and red asterisks indicate the conserved amino acids in the C-II-a (active) peptide; red asterisks indicate amino acids that were mutated in the C-II-i (inactive) peptide. (B) At 30 h after injection of 5 nl of C-II-a or C-II-i, at 6.5 dpf, larvae were stained with BODIPY. Red dextran fluorescence indicates successful injection. Scale bar: 50 µm. (C) Quantification of BODIPY staining results. Results are mean±s.e.m.; *n*=3 in each group; ***P<0.001; ns, not significant (Student's *t*-test). (D,E). TG and TC levels in homogenates of 6.5 dpf WT and *apoc2* mutant larvae, normalized to total protein content. Results are mean±s.e.m.; five embryos pooled per sample, *n*=3 in each group; *P<0.05; **P<0.01; ns, not significant (Student's *t*-test).

We have recently developed a novel bihelical amphipathic peptide (C-II-a) that contains an amphipathic helix (18A) for binding to lipoproteins and stimulating cholesterol efflux, as well as a motif based on the last helix of human APOC2, which activates lipolysis by LPL (Amar et al., 2015). An inactive APOC2 peptide (C-II-i), in which four amino acids are mutated, was used as a negative control. The C-II-a peptide restored normal lipolysis in plasma from APOC2-deficient human patients and dramatically decreased the TG levels in *Apoe^−/−^* mice (Amar et al., 2015). Protein alignment indicated that nine of 20 amino acids in C-II-a were conserved between zebrafish and human, including the crucial amino acids that were mutated in C-II-i (Fig. 6A). To test whether the human APOC2-based mimetic peptide could rescue hypertriglyceridemia in *apoc2* mutant zebrafish, we injected C-II-a or C-II-i, together with a fluorescent dextran tracer, into zebrafish larvae and assessed the vascular content of neutral lipids (using BODIPY staining) at 30 min and 6, 30 and 72 h after injection. Although at 30 min there was no difference, at 6 h post C-II-a injection, there was a modest, but statistically significant, 20% decrease in BODIPY fluorescence (supplementary material Fig. S4). Furthermore, at 30 h, the BODIPY signal was dramatically decreased by 60% in C-II-a-injected, but not in C-II-i-injected, zebrafish (Fig. 6B,C), and the effect of the C-II-a peptide persisted for at least 72 h (supplementary material Fig. S4). Biochemical measurements confirmed a significant decrease in TG levels and a trend towards TC decrease (Fig. 6D,E).

### Vascular lipid accumulation in *apoc2* mutant larvae

**Fig. 7.**
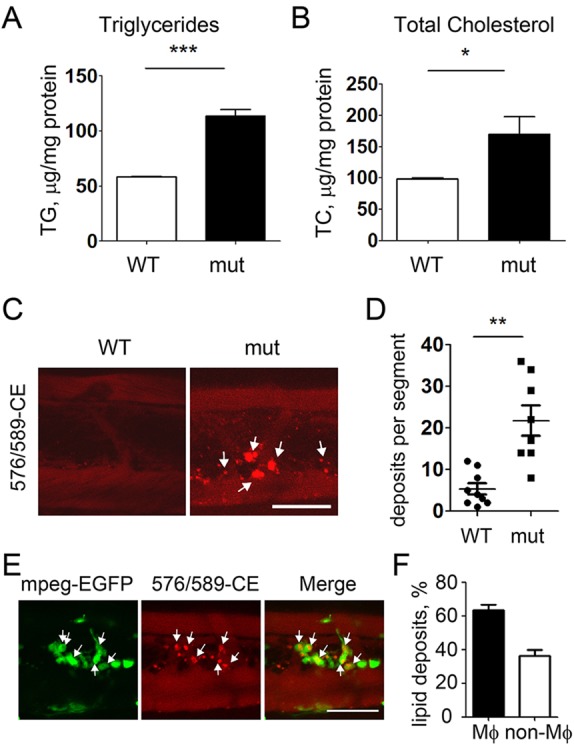
**Enhanced vascular lipid accumulation in *apoc2* mutants.** (A,B) TG and TC levels in homogenates of 14 dpf WT and *apoc2* mutant (mut) larvae, normalized to total protein content. Larvae were fed a normal diet. Results are mean±s.e.m.; *n*=3 in each group; *P<0.05; ***P<0.001 (Student's *t*-test). (C,D) Vascular lipid deposits in 14 dpf WT (*n*=8) and *apoc2* mutant (*n*=8) larvae fed a normal diet; **P<0.01 (Student's *t*-test). (E) Colocalization of vascular lipid deposits with macrophages in 14 dpf *apoc2* mutant *mpeg1:EGFP* zebrafish. (F) Quantification of lipid deposits associated and non-associated with macrophages in *apoc2* mutant larvae. Results are mean±s.e.m.; *n*=7. Scale bars: 50 µm.

In our previous experiments, we found that supplementing a high-cholesterol diet (HCD) with red fluorescent CE enabled monitoring accumulation of vascular lipid deposits in live transparent zebrafish larvae (Fang et al., 2011; Stoletov et al., 2009). As *apoc2* mutant zebrafish have higher TG and TC levels even when fed a diet with normal content of cholesterol (Figs 2, 4; Fig. 7A,B), we tested the *apoc2* mutants for the presence of vascular lipid deposits. WT and *apoc2* mutants were fed a diet with a normal content of fat and cholesterol from 5 to 14 dpf. To trace lipid accumulation, fluorescently labeled CE (576/589-CE; the numbers indicate excitation/emission wavelengths) was added to the diet from 8 to 12 dpf. The number of fluorescent lipid deposits was significantly higher in the vasculature of *apoc2* mutants than in WT zebrafish (Fig. 7C,D). To test whether these vascular lipid deposits were in fact intracellular lipid accumulated in macrophages, as observed in human and mouse early atherosclerotic lesions, we crossed *apoc2* mutants to *mpeg1-EGFP* transgenic zebrafish, which express EGFP in macrophages. As expected, the majority of the lipid deposits were localized within macrophages (Fig. 7E,F).

### Pancreatic ectopic growth in *apoc2* mutants

**Fig. 8.**
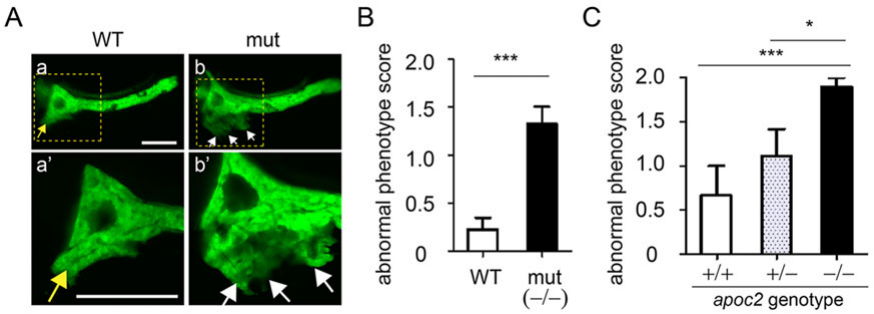
***apoc2* mutants have ectopic pancreas outgrowth.** (A) Images of pancreas, labeled with EGFP driven by the *ptf1a* promoter, in 6.5 dpf WT and *apoc2* mutant (mut) larvae. The yellow arrow points to the normal pancreatic growth and white arrows point to ectopic outgrowth. Scale bar: 100 µm. (B,C) At 6.5 dpf, an independent observer scored a pancreatic outgrowth phenotype as 0, normal, 1, mild and 2, severe. (B) WT (*n*=13) and *apoc2^−/−^* mutant (*n*=21). Results are mean±s.e.m.; ***P<0.001 (Student's *t*-test). (C) Heterozygous *apoc2^+/−^* zebrafish carrying one copy of the *ptf1a-EGFP* transgene were crossed with the heterozygous *apoc2^+/−^* zebrafish carrying no *ptf1a-EGFP*. A total of 22 embryos that displayed EGFP fluorescence were selected, scored for the pancreas outgrowth defect and then genotyped. Random *ptf1a-EGFP* expression resulted in the following distribution of EGFP-positive embryos: *apoc2^+/^*^+^ (*n*=3), *apoc2^+/−^* (*n*=9) and *apoc2^−/^*^−^ (*n*=10). Results are mean±s.e.m.; *P<0.05; ***P<0.001 (Student's *t*-test).

Patients with extremely high TG levels are at greater risk for the development of acute pancreatitis (Ewald et al., 2009). However, the mechanisms by which this occurs are not known with certainty, although they might be related in part to excess TG accumulation in the pancreatic vasculature, where they would be subjected to hydrolysis by pancreatic lipases (Wang et al., 2009). To assess developmental pancreatic defects, we crossed *apoc2* mutants with *ptf1a-EGFP* transgenic zebrafish, which express EGFP primarily in acinar cells (Dong et al., 2008; Godinho et al., 2005). We observed ectopic outgrowth of the acinar pancreas in *apoc2* mutants starting at 4 dpf, which was persistent and was also observed in 6.5 dpf embryos (Fig. 8A,B). At 6.5 dpf, a WT pancreas usually has a head domain, which contains the principal islet, and a long tail domain that extends posteriorly. In contrast, the *apoc2* mutant pancreas had additional short ectopic protrusions along the head of the pancreas (Fig. 8). These defects found in embryos from *apoc2^−/−^* incrosses were confirmed in *apoc2^+/−^* incross experiments in which embryos were scored for ectopic pancreatic outgrowth prior to genotyping (Fig. 8C).

## DISCUSSION

Recent genome-wide association studies provide new strong evidence and support earlier studies suggesting that abnormal metabolism of TRLs contributes to increased risk of CVD (Do et al., 2015, 2013; Hokanson and Austin, 1996). In addition, it has been shown that carriers of *APOC3* loss-of-function mutations have 40% lower plasma TG levels and a 40% lower risk of CVD than the general population (Jørgensen et al., 2014; The TG and HDL Working Group of the Exome Sequencing Project, National Heart, Lung, and Blood Institute et al., 2014). These two Mendelian randomization studies categorized participants based on their *APOC3* genotype rather than plasma TG levels, theoretically excluding confounding factors that affect both plasma TG levels and CVD (Cohen et al., 2014). Although one still cannot exclude the possibility that APOC3 plays its role in CVD through other pathways independent from its effects on TG levels, those two studies clearly link the plasma TG levels with the genetic cause of CVD risk.

A major mechanism by which APOC3 increases plasma TG is by its ability to inhibit LPL activity (Larsson et al., 2013), although it also is known to inhibit the clearance of VLDL and chylomicron remnants. In contrast, APOC2 is a cofactor of LPL and its loss of function results in deficient LPL activity and consequent hypertriglyceridemia and chylomicronemia (Breckenridge et al., 1978; Cox et al., 1978). In this work, we report the development of a new zebrafish model in which a loss-of-function mutation in the *apoc2* gene results in chylomicronemia and profound hypertriglyceridemia. The first *apoc2* mutant we selected had a frame shift mutation, which led to the amino acid sequence change in its Lpl-binding domain (Fig. 1B,E). In a different *apoc2* mutant line, we found a stop codon mutation, which caused deletion of the Lpl-binding domain from the Apoc2 protein sequence (supplementary material Fig. S3). Importantly, both *apoc2* mutant zebrafish lines showed dramatically decreased *apoc2* mRNA expression and developed hypertriglyceridemia, validating the Apoc2 loss-of-function model.

The plasma of adult *apoc2* mutant zebrafish shared many characteristics with the plasma of APOC2- or LPL-deficient human patients (Cox et al., 1978; Hooper et al., 2014; Okubo et al., 2014). It has significantly decreased lipase activity, chylomicronemia, dramatic hypertriglyceridemia and increased VLDL content, hypercholesterolemia and reduced levels of HDL (Fig. 2). The advantages of using zebrafish as an animal model include the optical transparency of embryos and larvae, which makes them suitable for live high-resolution imaging, and easy genetic manipulation and drug screening. Our studies demonstrate that as early as at 3 dpf, *apoc2* mutant larvae already display remarkable hyperlipidemia, and in 6 dpf embryos, dramatic differences in circulating neutral lipids were detected with ORO staining and confirmed with biochemical measurements of TG and TC levels in whole-body homogenates (Fig. 4).

In human APOC2 patients, chylomicron and VLDL size and abundance fluctuate widely and depend on the type of food consumed on the preceding day (Breckenridge et al., 1978). Future work will determine whether variation of the diet fed to *apoc2* mutant zebrafish will help maximize chylomicron or VLDL content for specific experimental goals. An important difference between mammalian and fish lipoprotein metabolism is the absence of APOBEC1 in non-mammalian vertebrates (Conticello et al., 2005; Teng and Davidson, 1992) and, thus, the absence of intestinal apoB-48, which is a major apolipoprotein in human chylomicrons. This might explain why the majority of TRLs we observed in *apoc2* mutant zebrafish were a similar size to human and mouse VLDL. The lipoprotein profiles shown in Fig. 2D-F suggest that the *apoc2* mutant zebrafish are suitable for modeling human dyslipidemia, and the zebrafish model favorably compares with a large number of other models, including the *Ldlr^−/−^* mouse, as profiled previously (Yin et al., 2012).

In this work, we developed a simple and convenient protocol for staining circulating lipoproteins with BODIPY. BODIPY is a lipophilic fluorescent dye, which preferentially localizes to the neutral lipid core of lipoproteins and intracellular lipid droplets, and is widely used in cell biology (Bozaquel-Morais et al., 2010; Gocze and Freeman, 1994; Grandl and Schmitz, 2010). However, to the best of our knowledge, BODIPY has not been used to stain and monitor circulating lipid levels in live zebrafish larvae. In our experiments, live zebrafish were immersed in a BODIPY solution, with a subsequent wash in water. This simple procedure resulted in bright green fluorescent staining of neutral lipids in the circulation of *apoc2* mutant animals. This assay provides a robust readout and might help conduct genetic and drug screening studies.

We used the BODIPY assay to demonstrate that injections of normal zebrafish plasma significantly diminished hypertriglyceridemia in *apoc2* mutant zebrafish (Fig. 5). This result is similar to the treatment of APOC2-deficient patients with severe hypertriglyceridemia in which transfusions of normal, APOC2-containing plasma leads to rapid (within 1 day) reductions in plasma TGs (Breckenridge et al., 1978). Remarkably, the amino acids that are crucial for LPL activation in the last helix of human APOC2 are conserved in zebrafish, and the human APOC2 mimetic peptide C-II-a (Amar et al., 2015), rescued severe hypertriglyceridemia in *apoc2* mutant zebrafish (Fig. 6). These results suggest that our zebrafish model is suitable for testing potential therapeutic agents for treatment of human APOC2 deficiency.

Two previous studies in which *apoc2* was transiently knocked down in zebrafish embryos with antisense morpholino oligonucleotides, have suggested that Apoc2 is involved in yolk absorption and angiogenesis (Avraham-Davidi et al., 2012; Pickart et al., 2006). Our results with the *apoc2* mutant zebrafish support these conclusions. Consistent with the results reported by Pickart et al., *apoc2* mutant larvae show a slightly delayed yolk consumption (Fig. 3A-G) and the mutant larvae are smaller than WT larvae. This phenotype in the *apoc2* mutant can be explained, in part, by the disruption of the Lpl function, resulting in a defect in TG hydrolysis and the supply of free fatty acids (FFAs) to non-hepatic tissues, which, in turn, might cause nutrient deprivation and delayed growth. In contrast, by monitoring the head angle at 28 hpf and the pattern of pigmentation at 3 dpf, we did not notice any differences in the stage of development reached between WT and *apoc2* mutants. Because adult *apoc2* mutant zebrafish are the same size as WT and have no apparent phenotype (Fig. 2A; supplementary material Fig. S1), other than hyperlipidemia, it is possible that feeding and the activity of other lipases help restore the energy balance in unchallenged animals.

A previous study has reported that a transient *apoc2* knockdown by antisense morpholino oligonucleotides increases levels of apoB-containing lipoproteins, which, in turn, inhibits embryonic angiogenesis (Avraham-Davidi et al., 2012). In our experiments, the Apoc2 loss of function resulted in dramatically increased levels of VLDL, an apoB-containing lipoprotein, and indeed delayed ISV and SIV growth. Nevertheless, the *apoc2* mutants were viable and their vascular structure was normal at 14 dpf despite profound hyperlipidemia (supplementary material Fig. S2). Thus, the excess of apoB-containing lipoproteins is likely to delay but not to completely inhibit angiogenesis *in vivo*.

In agreement with epidemiologic studies suggesting that hypertriglyceridemia is a risk factor for CVD (Do et al., 2015; Jørgensen et al., 2014; The TG and HDL Working Group of the Exome Sequencing Project, National Heart, Lung, and Blood Institute et al., 2014), our results demonstrate that the hypertriglyceridemia induced by the *apoc2* mutation in zebrafish results in vascular lipid accumulation and macrophage lipid uptake (Fig. 7C-E), which are important initial events in the development of human atherosclerosis. The vascular lipid accumulation in zebrafish induced by hypertriglyceridemia was similar to that induced by hypercholesterolemia (O'Hare et al., 2014; Stoletov et al., 2009). Our study is also consistent with mouse studies in which hypertriglyceridemia induced by *Lpl* or *Gpihbp* knockout resulted in spontaneous development of atherosclerosis (Weinstein et al., 2010; Zhang et al., 2008). In contrast to hypertriglyceridemic mice, in which atherosclerotic lesions develop late, by the age of 1 year, vascular lipid deposits in *apoc2* mutant zebrafish fed normal diet developed as early as at 14 dpf. One possible explanation is the absence of CETP in mice, whereas zebrafish express functional *Cetp* (Jin et al., 2011; Kim et al., 2012). Transfer of cholesterol from HDL to LDL and VLDL (and TG in the opposite direction) might play an atherogenic role in *apoc2* mutant zebrafish, as it does in humans (Barter and Rye, 2012).

One important clinical complication of hypertriglyceridemia in patients with LPL or APOC2 deficiency is recurrent pancreatitis, but the underlying mechanisms are poorly defined (Baggio et al., 1986; Fellin et al., 1983). In *apoc2* mutant zebrafish, we found ectopic acinar outgrowth in the head of the pancreas (Fig. 8). Acinar cells express high levels of pancreatic lipase. Chylomicronemia results in increased FFA uptake and injury in the acinar cells, which might lead to inflammation and pancreatitis (Scherer et al., 2014; Wang et al., 2009). Our findings suggest that the Apoc2 deficiency affects early pancreas development in zebrafish. A recent report shows that modeling chylomicron retention disease in zebrafish, which manifests in the reduction of circulating neutral lipids, was associated with inhibited growth of exocrine pancreas (Levic et al., 2015). We as yet do not know whether the hyper- or hypo-triglyceridemia effects on exocrine pancreas growth in *apoc2* mutant zebrafish are related to the pathogenesis of pancreatitis in human APOC2-deficient patients.

In summary, our new *apoc2* mutant zebrafish display a robust hyperlipidemia phenotype and present a useful and versatile animal model to study mechanisms related to human diseases induced by hypertriglyceridemia. Small-molecule and genetic screens using the *apoc2* mutant zebrafish might suggest new approaches to treatment of hyperlipidemia and related disorders.

## MATERIALS AND METHODS

### Zebrafish maintenance and feeding

Adult zebrafish of the AB strain were maintained at 28°C on a 14-h-light–10-h-dark cycle and fed brine shrimp twice a day. Zebrafish larvae were fed Golden Pearls (100–200 µm size from Brine Shrimp Direct, Ogden, UT) twice a day, starting from 4.5 dpf. For lipid-deposition experiments, Golden Pearls, supplemented with 1 µg/g of a fluorescent cholesterol ester analog (cholesteryl BODIPY 576/589-C11 from Invitrogen, Carlsbad, CA; catalog number C12681), were fed to zebrafish from 8 to 12 dpf as described previously (Stoletov et al., 2009). *fli1:EGFP* and *mpeg1*-EGFP transgenic zebrafish were from the Weinstein (Lawson and Weinstein, 2002) and Lieschke (Ellett et al., 2011) laboratories, respectively. *ptf1a*-EGFP transgenic zebrafish were developed in the Dong laboratory (Dong et al., 2008; Godinho et al., 2005). All animal studies were approved by the UCSD Institutional Animal Care and Use Committee.

### TALEN construction, mRNA synthesis, injection and confirmation of *apoc2* mutation

TALEN plasmids targeting *apoc2* were constructed from the stock cassette according to the published protocols (Dong et al., 2011; Huang et al., 2014). TALEN mRNAs were synthesized with an mMESSAGE mMACHINE Sp6 transcription kit (Ambion, Austin, TX; AM1340). mRNAs encoding the pair of TALEN proteins were mixed at a 1:1 ratio to a final concentration of 200 ng/µl. A total of 1-2 nl of TALEN mRNAs was injected into the one-cell stage embryos. Genomic DNA (gDNA) was extracted from whole embryos or from the tissue clipped off the tail fin of adult zebrafish and was genotyped by PCR (with the following primers: forward, 5′-GTGGTCGCATAGTTTAAGCT-3′; reverse, 5′-GCAACCATGTAAGAGTTGCA-3′) and BtsI enzyme digestion, and confirmed by sequencing, according to published protocols (Dong et al., 2011; Huang et al., 2014). To assess expression of *apoc2* in larvae homogenates and in adult liver, total RNA was isolated, reverse transcribed and subjected to qPCR with the following primers: forward, 5′-ATGAACAAGATACTGGCTAT-3′; reverse, 5′-TTGATGGTCTCTACATATCC-3′, using a KAPA SYBR FAST Universal qPCR kit (KAPA Biosystems, Wilmington, MA; KK4602) and a Rotor Gene Q qPCR machine (Qiagen, Valencia, CA). The position of these qPCR primers relative to the TALEN target site is shown in supplementary material Fig. S3. Because the mutation does not change nucleotide sequence beyond the target site, these primers detect expression of both WT and mutant mRNA, if any is present.

### Oil red O staining and BODIPY staining

Oil red O (ORO) staining was conducted according to published protocols (Schlegel and Stainier, 2006). Briefly, embryos were fixed in 4% paraformaldehyde (PFA) for 2 h, washed three times in PBS, incubated in 0.3% ORO solution for 2 h and then washed with PBS before imaging. For BODIPY staining, live larvae were immersed in E3 water (5.0 mM NaCl, 0.17 mM KCl, 0.33 mM CaCl_2_, 0.33 mM MgSO_4_) containing 0.1 µg/ml BODIPY^®^ 505/515 (4,4-difluoro-1,3,5,7-tetramethyl-4-Bora-3a,4a-diaza-*s*-indacene, Invitrogen; D-3921) for 1 h in dark and then rinsed with E3 water before imaging.

### Triglyceride and cholesterol measurements and lipoprotein analysis

Blood was collected from adult male zebrafish, after overnight fasting, through tail amputation and diluted 1:50 (WT) or 1:200 (*apoc2* mutant) in PBS. The supernatants were collected as a plasma fraction after centrifugation at 2350 ***g*** for 10 min. Embryos or larvae were gently homogenized in PBS with a pestle. After centrifugation at 16,000 ***g*** for 10 min, supernatants were collected and referred to as ‘homogenates’. TG and TC levels were measured in 25 µl of diluted plasma or embryo or larva homogenates using kits from BioVision (Milpitas, CA; Triglyceride Quantification Kit, K622-100; Cholesterol Quantification Kit, K623-100) and according to the manufacturer's protocol. To assess undiluted plasma, 5 µl blood was collected in a tube containing 0.5 µl heparin (5 mg/ml) and then loaded into a heparin-rinsed capillary, which was kept overnight at 4°C in a vertical position. Following erythrocyte sedimentation, the capillaries were photographed. Lipoprotein fractions were assessed using native agarose gel electrophoresis (Helena Laboratories, Beaumont, TX; 3045) as we previously described (Stoletov et al., 2009).

### Fast protein liquid chromatography lipoprotein profile

A total of 100 µl of pooled plasma from 45 adult WT zebrafish or 40 µl of pooled plasma from 14 adult *apoc2* mutant zebrafish were loaded onto a Superose 6 PC 3.2/30 column (GE Healthcare Life Science, Pittsburgh, PA; 17-0673-01), and TC and TG levels were determined in each fraction (250 μl), collected at a flow speed of 0.5 ml/min.

### Ultracentrifugation and electron microscopy

Pooled plasma from fasted overnight WT and *apoc2* mutant zebrafish was diluted in PBS, without density adjustment, and the samples were centrifuged at 200,000 ***g*** for 4 h at 4°C. The top layer containing TRLs was collected, supplemented with 0.1% sucrose, added to a FCF100-Cu 100 mesh (Electron Microscopy Sciences, Hatfield, PA) and negatively stained with 1% uranyl acetate. Stained samples were imaged with a Tecnai G2 Spirit BioTWIN transmission electron microscope equipped with a 4K Eagle digital camera (FEI Company, Hillsboro, OR).

### Plasma lipase activity assay

Adult zebrafish blood was diluted 1:25 in PBS and after centrifugation, 5 µl of the diluted plasma was used to measure lipase assay with a Lipase activity assay kit (Cayman Chemical, Ann Arbor, MI; 700640), following the manufacturer's manual. The assay buffer was used as a negative control. The reaction was conducted at 30°C.

### Intravenous injections

Blood was collected by tail amputation from adult male zebrafish at 4 h after the last feeding, and plasma was separated by centrifugation at 2350 ***g*** for 10 min. C-II-a and C-II-i peptides (Amar et al., 2015) were dissolved in PBS (pH 7.4) to a concentration of 2 mg/ml. To ensure visual control of successful injection, zebrafish plasma or APOC2 mimetic peptides were supplemented with 2% (v/v) tetramethylrhodamine-conjugated 10 kDa dextran (0.5 mg/ml miniRuby, Invitrogen, D-3312). For injections, 5 dpf larvae were aligned in 0.5% low-melting-point agarose. A total of 5 nl of plasma or APOC2 mimetic peptides were injected through the cardinal vein above the heart chamber using a FemtoJet microinjector (Eppendorf, Hamburg, Germany). The red signal from fluorescent dextran confirmed successful injection.

### Imaging of live embryos or larvae

For *in vivo* live microscopy, anesthetized embryos or larvae were mounted in low-melting-point agarose (0.5%, Fisher, Pittsburgh, PA; BP1360-100) containing tricaine (0.02%, Sigma, St Louis, MO; A5040) in 50-mm glass-bottom dishes (MatTek, Ashland, MA; P50G-0-14-F). Images were captured with Leica CTR5000 (Wetzlar, Germany), Olympus FV1000 spectral confocal (Tokyo, Japan) or BZ9000 Keyence (Osaka, Japan) fluorescent microscopes.

## Supplementary Material

Supplementary Material
