# Supplementary material for: Apoc2 loss-of-function zebrafish mutant as a genetic model of hyperlipidemia
Source: Dis Model Mech. 2015 Aug 1;8(8):989–98. doi: 10.1242/dmm.019836 (PMC4527288; doi:10.1242/dmm.019836)
Supplement: Supplementary Material [file supp_8_8_989__index.html]

Supplementary Material 

# Apoc2 loss-of-function zebrafish mutant as a genetic model of hyperlipidemia

## DMM019836 Supplementary Material

- Supplementary Material
