# Supplementary material for: Apoc2 loss-of-function zebrafish mutant as a genetic model of hyperlipidemia
Source: Dis Model Mech. 2015 Aug 1;8(8):989–98. doi: 10.1242/dmm.019836 (PMC4527288; doi:10.1242/dmm.019836)
Supplement: Supplementary Material [file supp_019836_DMM019836supp.pdf]

Liu et al. Apoc2 loss-of-function zebrafish mutant as a genetic model of hyperlipidemia

## Supplementary Figures

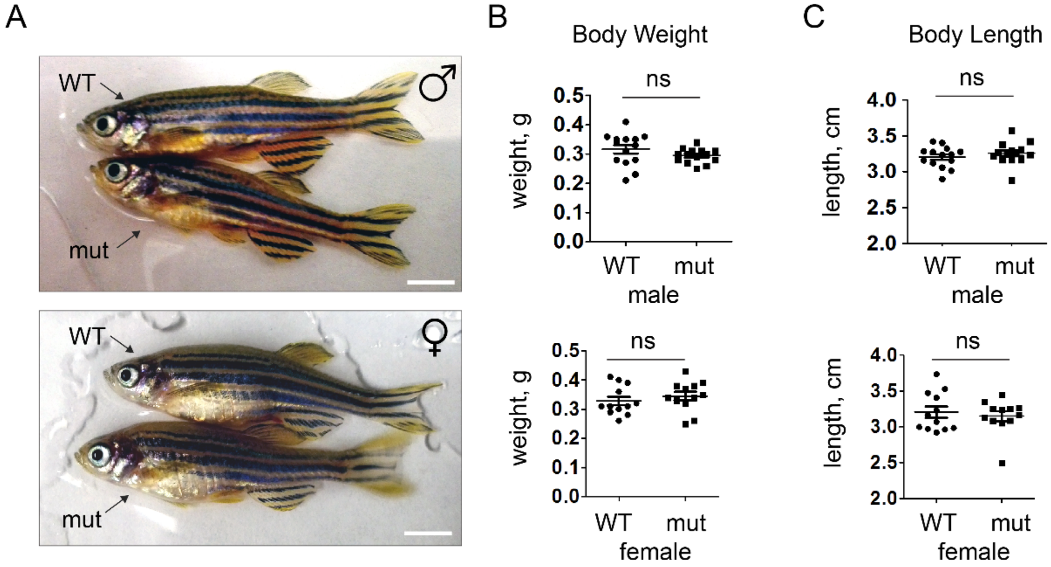


**Figure S1: Body weight and size of adult WT and *apoc2* mutant zebrafish.** Physical appearance (A) and body weight and length (B) of WT (n=14) and mutant (n=14), 10 month old, male and female zebrafish. Scale bar, 0.5 cm.

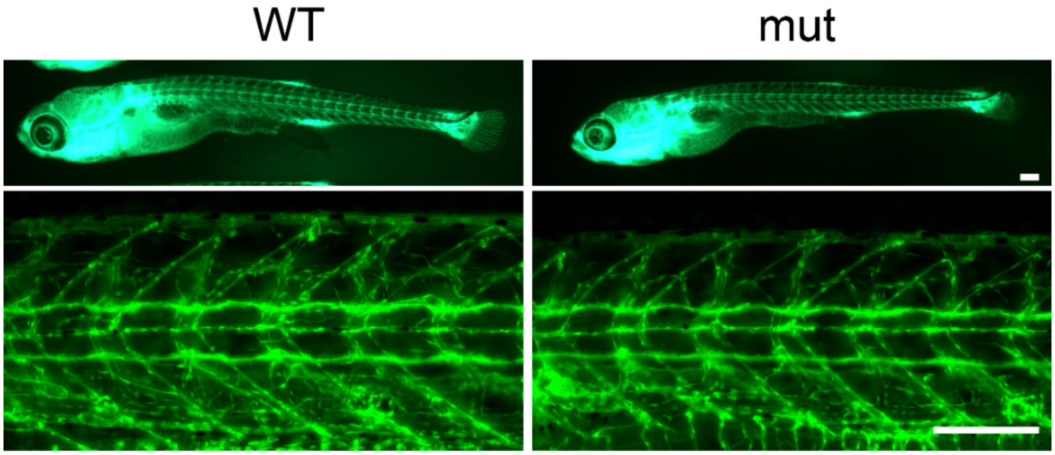


**Figure S2: Angiogenesis in *fli1:EGFP* WT and *apoc2* mutant zebrafish at 14 dpf**

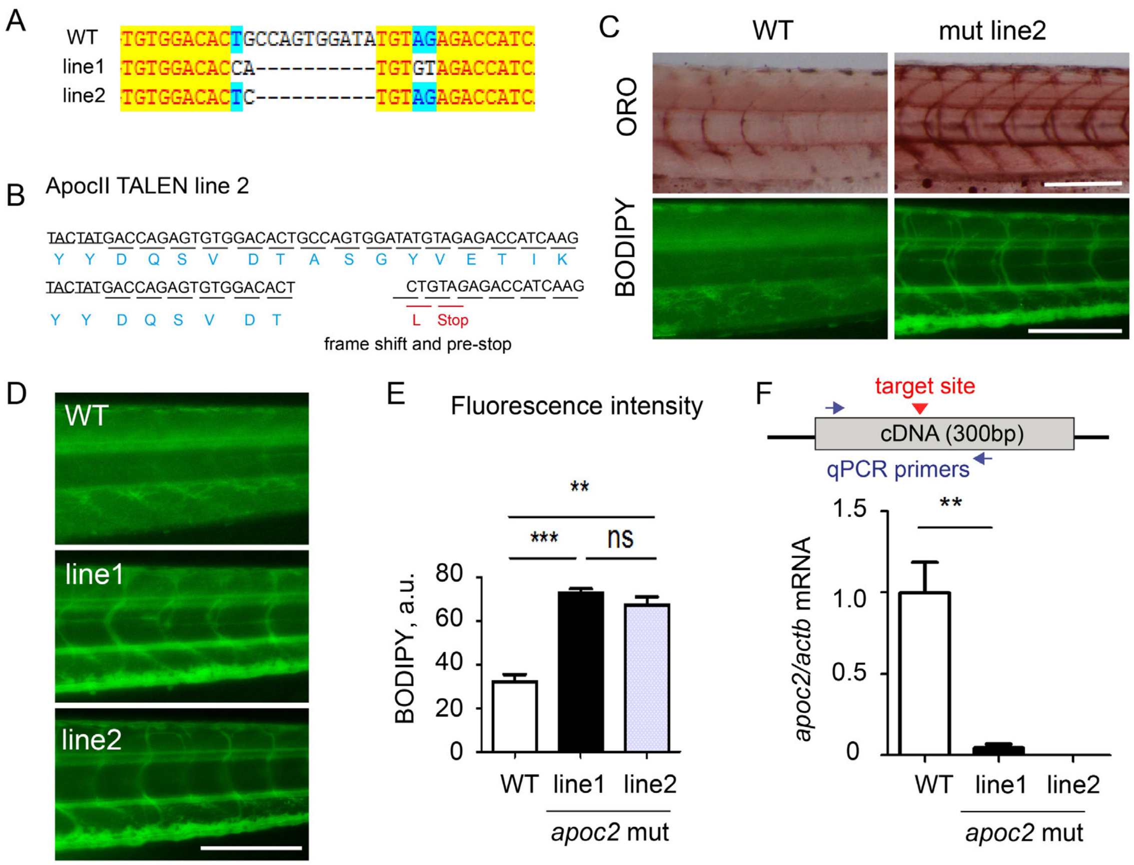


**Figure S3: Additional *apoc2* mutant zebrafish line**

(A) Sequence results of the *apoc2* genomic DNA from the mutant line 1 (shown in Fig. 1) and the additional line 2. (B) Mutation in line 2 introduced a stop codon in the *apoc2* gene. (C) BODIPY and ORO staining of WT and line 2 *apoc2* mutant at 6 dpf. Scale bar, 200 µm. (D) BODIPY staining of WT and line 1 and line apoc2 mutant larvae at 6 dpf. Scale bar, 200 µm. (E) Quantification of BODIPY staining results shown in panel D. Mean±SEM; n=3 in each group; ***, p<0.001; **, p<0.01. (F) The diagram shows the position of qPCR primers relative to the mutation target site. *apoc2* mRNA expression (qPCR) in whole body homogenates of 5.5 dpf WT, mutant line 1 and mutant line 2 larvae. Mean±SEM; n=3 in each group; **, p<0.01. (Part of this graph is also shown in Fig. 1G.)

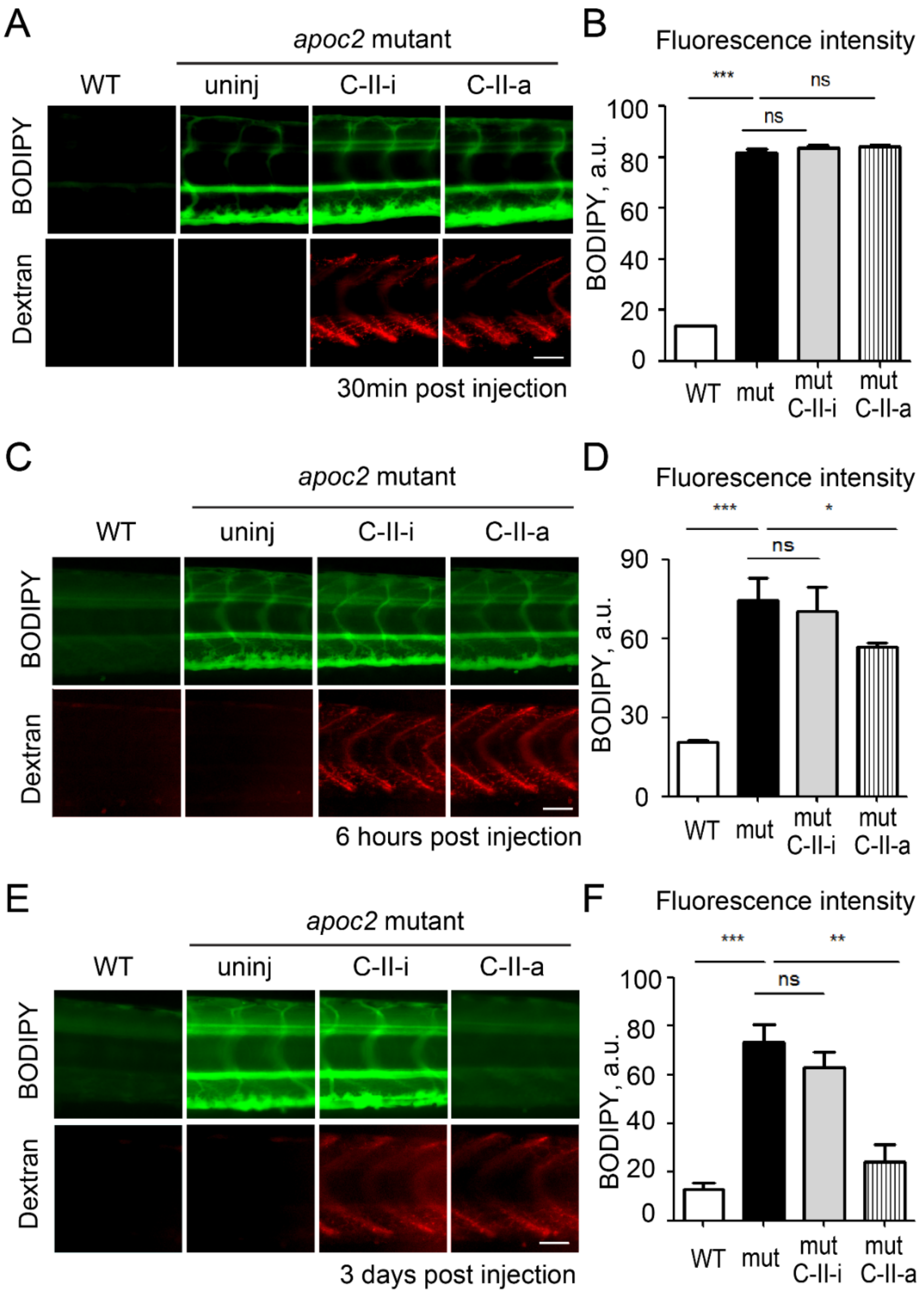


**Figure S4: Injection of human apoC-II mimetic peptide: Time course**
The C-II-a and C-II-i peptides were injected as in Fig. 6. (A and B) 30 minutes after injection; (C and D) 6 hours after injection; (E and F) three days after injection. Larvae were stained with BODIPY. Red dextran fluorescence indicates successful injection. Scale bar, 50 µm. (B, D and F) Quantification of BODIPY staining results. Mean±SEM; n=3; ***, p<0.001; **, p<0.01; *, p<0.05; ns, not significant.
